# Clinical inflammatory shifts and peripheral flow-cytometry immune modules across benign pulmonary nodules, adenocarcinoma-spectrum nodules, and overt lung cancer

**DOI:** 10.3389/fimmu.2026.1920402

**Published:** 2026-09-01

**Authors:** Yunwei Rao, Qianyue Gong, Jiawei Li, Nan Liu, Hongmei Miao, Jinyue Huang, Shuiming Xu

**Affiliations:** 1 Department of Respiratory and Critical Care Medicine, First Affiliated Hospital of Gannan Medical University, Ganzhou, Jiangxi, China; 2 Medical School, Shaoguan University, Guangzhou, Guangdong, China; 3 Institute of Biomedical Translational Research, Jinan University, Guangzhou, Guangdong, China; 4 School of Public Health and Health Management, Gannan Medical University, Ganzhou, Jiangxi, China; 5 Guangdong Provincial People’s Hospital Ganzhou Hospital (Ganzhou Municipal Hospital), Ganzhou, Jiangxi, China; 6 School of Clinical Medicine, Nanchang Medical College, Nanchang, Jiangxi, China

**Keywords:** clinical inflammation, exploratory cohort, flow cytometry, immune module, lung cancer, lymphocyte axis, peripheral immunity, pulmonary nodule

## Abstract

**Objective:**

Pulmonary nodules (PN) represent a clinically heterogeneous pathway that includes benign lesions, adenocarcinoma-spectrum lesions, and cases that may differ from clinically overt lung cancer (LC). This study aimed to define a peripheral blood immunophenotyping framework for this pathway by integrating routine inflammatory readouts with transparently defined whole-blood flow-cytometry immune modules.

**Methods:**

We performed a retrospective analysis of a single-center locked cohort of 217 participants, including healthy controls (n=121), pulmonary nodules (n=46), and overt lung cancer (n=50). The PN group was further stratified into benign PN (n=20) and adenocarcinoma-spectrum PN (n=26). Clinical readouts included neutrophil-to-lymphocyte ratio (NLR), systemic immune-inflammation index (SII), albumin, and lymphocyte measures. Peripheral immune phenotypes were summarized using four code-defined flow-cytometry module scores: B-cell remodeling, Tfh/Th2 skew, cytotoxic attenuation, and CD28-loss. Group differences were evaluated using Cliff’s delta with 95% confidence intervals and FDR-adjusted q values, with adjusted models used only as supportive diagnostics.

**Results:**

Within the PN cohort, adenocarcinoma-spectrum PN showed a coherent inflammatory and lymphocyte-axis shift relative to benign PN, including higher NLR (delta=0.70, q<0.001), higher SII (delta=0.62, q<0.001), higher inflammation score (delta=0.81, q<0.001), lower albumin (delta=-0.69, q<0.001), and lower lymphocyte percentage (delta=-0.72, q<0.001). In the peripheral immune layer, overt LC showed higher module scores than PN across B-cell remodeling (delta=0.38, q=0.002), Tfh/Th2 skew (delta=0.40, q=0.001), cytotoxic attenuation (delta=0.47, q<0.001), and CD28-loss (delta=0.27, q=0.021). Focused and sensitivity comparisons preserved the direction of the module findings, with the strongest support for cytotoxic attenuation and Tfh/Th2 skew.

**Conclusion:**

This study provides a clinically anchored description of peripheral inflammatory and immune patterns across clinically defined PN and overt LC groups. The findings identify inflammatory heterogeneity within PN and higher immune-module scores in overt LC, providing a hypothesis-generating framework for prospective validation and paired tissue-peripheral studies.

## Introduction

Pulmonary nodules are encountered frequently in clinical practice, especially with increased use of chest computed tomography and structured surveillance pathways (1–3). Although many nodules are benign, a subset represents adenocarcinoma-spectrum lesions or early invasive disease. This mixture creates a practical problem for translational studies: the term pulmonary nodule does not describe a single biological state. A pulmonary nodule cohort may include benign inflammatory or non-neoplastic conditions, atypical adenomatous hyperplasia, adenocarcinoma *in situ*, minimally invasive adenocarcinoma, and small invasive adenocarcinoma that is detected and managed through the pulmonary-nodule clinical pathway (4, 5). Overt lung cancer cohorts are often clinically distinct because they include patients managed through a lung-cancer pathway, with more advanced pathology, different clinical context, and potentially different systemic immune or inflammatory status.

Most clinical and translational analyses of pulmonary nodules focus on imaging characteristics, histology, or clinical classification. Peripheral blood data are often treated as secondary covariates, even though routine clinical readouts such as neutrophil-to-lymphocyte ratio, monocyte-to-lymphocyte ratio, systemic immune-inflammation index, albumin, absolute lymphocyte count, and lymphocyte percentage can capture broad inflammatory, nutritional, and immune-cell availability signals. These variables are not specific markers of lung cancer, although NLR, MLR, SII, albumin-related measures, and composite nutritional-inflammatory indices have been widely studied as nonspecific systemic readouts in cancer and lung-cancer cohorts (6–12). However, when interpreted carefully and in combination, they can help describe whether adenocarcinoma-spectrum nodules already differ from benign nodules at the level of systemic clinical readouts. Such a description is particularly useful when it is separated from diagnostic claims and framed as a cohort-level association.

Peripheral immune phenotyping provides a second layer of information. Flow cytometry can capture cellular phenotypes that are not directly visible in routine blood tests, but it requires transparent reporting of gating strategy, reagents and analysis procedures (13, 14). In this study, we organized peripheral immune phenotypes into organize four code-defined module scores: B-cell remodeling, Tfh/Th2 skew, cytotoxic attenuation, and CD28-loss module. These module scores serve as compact summaries of related flow-cytometry immune phenotypes, including peripheral T-cell, NK-cell, gamma-delta T-cell, and B-cell marker-defined populations that have broader precedent in cancer immunophenotyping studies (15–29).

The primary objective was to compare cross-sectional inflammatory and peripheral immune patterns among benign PN, adenocarcinoma-spectrum PN, and overt LC rather than to develop a diagnostic model. We hypothesized that these clinically defined groups would differ in routine inflammatory readouts and flow-cytometry module scores. Our analytical strategy prioritized local flow -cytometry module scores, supported by clinical inflammatory markers. Public transcriptomic and tissue datasets were used solely for contextual interpretation because of differences in measurement platforms and tissue compartments.

## Materials and methods

### Study design and analysis boundary

This was an exploratory single-center retrospective locked-cohort analysis of peripheral clinical readouts and flow-cytometry immune phenotypes across HC, PN, and overt LC groups, structured with attention to observational-reporting and flow-cytometry reporting principles (30, 31). The study was conducted at The First Affiliated Hospital of Gannan Medical University, Ganzhou, Jiangxi, China, and was approved by the Scientific Research Ethics Committee of the First Affiliated Hospital of Gannan Medical University (approval number LLSC-2023 NO.273; approval date October 16, 2023). The ethics- approval project period was January 1, 2024 to December 31, 2026; The present analysis used the locked retrospective dataset available at the time of analysis. Data were de-identified before analysis.

The cohort comprised 217 participants: HC n=121, PN n=46, and overt LC n=50. The PN group was further divided into Benign PN n=20 and Adeno-spectrum PN n=26. HC were individuals with no known medical history and normal chest CT findings. PN were patients diagnosed with pulmonary nodules by chest CT. Overt LC was defined as pathologically confirmed primary lung cancer managed through the lung-cancer clinical pathway, with blood collected before anti-cancer treatment.

The group definitions were organized around clinical analysis pathways rather than a simple benign/malignant dichotomy. Benign PN represented benign non-neoplastic pulmonary nodules confirmed by pathology when available or by at least 6 months of clinical follow-up/adjudication. Adeno-spectrum PN represented pulmonary-nodule pathway cases with adenocarcinoma-spectrum pathology, including precursor or pre-invasive lesion, AIS, MIA, and small invasive adenocarcinoma presenting through the pulmonary-nodule clinical pathway. Overt LC comprised pathologically confirmed primary lung cancer managed through the lung-cancer clinical pathway. Participants with incomplete immune profiling or baseline demographic/clinical data, prior anti-cancer or immunomodulatory treatment, autoimmune disease, pregnancy or lactation, or acute or chronic active infection were excluded.

### Clinical readouts

The clinical readout layer focused on complete-case blood and chemistry variables. The main comparison contrasted Benign PN with Adeno-spectrum PN. NLR was calculated as absolute neutrophil count divided by absolute lymphocyte count; MLR as absolute monocyte count divided by absolute lymphocyte count; and SII as platelet count multiplied by absolute neutrophil count and divided by absolute lymphocyte count. The inflammation score was a code-defined composite calculated as the mean of population z-scored NLR, MLR, and SII minus the mean of population z-scored absolute lymphocyte count, albumin, and hemoglobin. Component z-scores were computed over the locked analysis cohort. Available components were averaged within each side with missing values ignored; if all components on either side were missing, the composite score was treated as missing.

### Flow cytometry phenotyping and gating support

Peripheral immune phenotypes were measured using a multiparameter flow-cytometry panel from whole blood. For each participant, 3 mL peripheral blood was collected in sodium heparin anticoagulant tubes and processed within 48 hours. Red blood cell lysis was performed using 1× red blood cell lysis buffer (TIANGEN) at room temperature (20–30 °C) for 10 minutes. PBMC isolation was not performed. For each staining tube, 100 μL whole blood was incubated with 10 μL antibody working solution prepared at 1:10 dilution, corresponding to a final dilution of 1:100, for 30 minutes at 4 °C in the dark. Samples were washed once with PBS and resuspended in 200 μL 1× PBS. Fixation and dedicated viability-dye staining were not performed. FSC-A/SSC-A gating was used to exclude debris; because viability was not assessed with a dedicated dye, residual nonviable-cell contamination cannot be excluded.

Data were acquired on a FACSLyric flow cytometer (BD Biosciences) configured with 488 nm, 640 nm, and 405 nm lasers using BD FACSuite acquisition software. Between 500,000 and 1,000,000 events were acquired per panel. Daily instrument quality control used CS&T calibration beads. Compensation matrices were derived from single-stained cell controls. FMO controls were used for threshold setting where available; thresholds for remaining markers were based on negative populations and a fixed laboratory gating template. Isotype controls were not used, and the same hierarchical gating template was applied across samples. Exact collection-to-acquisition times within the 48-hour window and flow-cytometry batch identifiers were not retained in the locked dataset and could not be modeled.

Flow-cytometry data were analyzed in FlowJo v10 (BD Biosciences) by three analysts under a blinded workflow using a unified hierarchical gating template. The antibody panel, marker-defined phenotype definitions, and denominator/unit reference are provided in the Supplementary Tables. Representative gates are shown in **Supplementary Figure 1** as methods documentation rather than group-comparison evidence. No minimum event threshold for individual parent gates was specified in the available study documentation.

### Module score construction

Four peripheral flow-cytometry module scores were analyzed: B-cell remodeling, Tfh/Th2 skew, cytotoxic attenuation, and CD28-loss module. The module components, directions, and aggregation rule were fixed in the analysis code used to generate the reported locked outputs. An independent preregistration or pre-comparison freeze record was not available; the modules are therefore described as code-defined rather than prespecified:

module score = mean(z-positive components) - mean(z-negative components).

Each component phenotype was transformed using population z-scoringover the locked full cohort before module aggregation. Positive and negative component means were calculated from the available component z-scores, with missing values ignored within each side. If all positive or all negative components were missing for a participant, the module score was treated as missing. Component directions, marker-defined phenotypes, and the aggregation rule are reported in **Supplementary Table 6**. The modules are analytical summaries of marker-defined phenotypes.

### Statistical analysis

Continuous variables were summarized as median [Q1, Q3], and categorical variables as n (%). The sample size was determined by all eligible records available in the locked retrospective cohort; no prospective power calculation or confirmatory effect-size target was specified. Precision was conveyed using bootstrap 95% confidence intervals. For the PN clinical readout comparison, Benign PN and Adeno-spectrum PN were compared. For the peripheral immune-module analysis, PN versus overt LC was the primary comparison, and Adeno-PN versus overt LC was a focused comparison. Sensitivity comparisons included PN versus NSCLC only, PN versus LC excluding stage IV, and Adeno-PN versus LUAD only.

Two-group P values were obtained from two-sided Wilcoxon rank-sum tests using the normal approximation (exact=FALSE). Effect sizes were summarized using Cliff’s delta (32), oriented as the second-listed group relative to the first-listed group. Where reported, 95% confidence intervals for Cliff’s delta were percentile bootstrap intervals based on 2,000 within-group resamples (random seed 20260503) (33). Benjamini-Hochberg FDR correction (34) was applied separately within each comparison-specific family: the nine clinical readouts in the PN comparison and the four module scores within each module contrast. Analyses used complete cases for each readout or score; no imputation was performed. Age- and sex-adjusted logistic regression models were fitted with binomial generalized linear models and were treated as supportive diagnostics. The standard logistic estimate for cytotoxic attenuation was not reported because possible separation was flagged (35, 36). Analyses were implemented in R 4.5.3 using base R and the stats package. Public transcriptomic resources were contextual only and were outside the primary FDR-controlled analyses.

## Results

### Cohort composition and pathology context

The cohort included 217 participants: HC (n=121), PN (n=46), and Overt LC (n=50). PN comprised Benign PN (n=20) and Adeno-spectrum PN (n=26). Median age was 58 years in HC, 52.5 years in Benign PN, 52.5 years in Adeno-spectrum PN, and 59 years in Overt LC; male participants accounted for 47.9%, 35.0%, 53.8%, and 54.0%, respectively (**Table 1**).

**Table 1:** Baseline characteristics of the study cohort.

Characteristic	HC (n=121)	Benign PN (n=20)	Adeno-spectrum PN (n=26)	Overt LC (n=50)
Total participants, n	121	20	26	50
Age, years	58 [49, 61]	52.5 [43, 56]	52.5 [49, 64.8]	59 [52, 68.8]
Sex: male, n (%)	58 (47.9%)	7 (35.0%)	14 (53.8%)	27 (54.0%)
Sex: female, n (%)	63 (52.1%)	13 (65.0%)	12 (46.2%)	23 (46.0%)
White blood cell count, ×10^9/L	5.93 [5.16, 6.9]	5.71 [5.09, 7.11]	6.79 [5.23, 8.63]	7.51 [5.39, 8.7]
Absolute neutrophil count, ×10^9/L	3.29 [2.88, 3.74]	3.24 [2.71, 3.75]	4.5 [3.58, 5.94]	5.31 [3.25, 6.41]
Absolute lymphocyte count, ×10^9/L	1.92 [1.65, 2.53]	1.91 [1.71, 2.4]	1.5 [1.05, 1.79]	1.41 [1.12, 1.84]
Hemoglobin, g/L	144 [135, 153]	140 [130, 150]	132 [115, 142]	126 [108, 135]
Platelet count, ×10^9/L	246 [196, 273]	294 [234, 317]	282 [247, 330]	257 [223, 354]
Albumin, g/L	46 [44.7, 46.9]	46 [45, 47.2]	39.7 [36, 43.2]	39.3 [37.2, 43]
CEA, ng/mL	2.4 [1.68, 3.08]	2.15 [1.34, 2.72]	2.53 [1.33, 4.16]	3.14 [1.96, 13.3]
PN pathology	—	—	—	—
Benign non-neoplastic	—	20 (100.0% of Benign PN)	—	—
Precursor / pre-invasive lesion	—	—	6 (23.1% of Adeno-spectrum PN)	—
AIS	—	—	7 (26.9% of Adeno-spectrum PN)	—
MIA	—	—	7 (26.9% of Adeno-spectrum PN)	—
Small invasive adenocarcinoma	—	—	6 (23.1% of Adeno-spectrum PN)	—
Overt LC histology	—	—	—	—
NSCLC	—	—	—	46 (92.0%)
SCLC	—	—	—	4 (8.0%)
Overt LC stage	—	—	—	—
Early	—	—	—	15 (30.0%)
Locally resectable / intermediate	—	—	—	9 (18.0%)
Locally advanced	—	—	—	13 (26.0%)
Metastatic / advanced	—	—	—	13 (26.0%)

1. Values are median [Q1, Q3] or n (%), unless otherwise indicated.

2. Available observations were: full cohort for age and sex; HC/Benign PN/Adeno-spectrum PN/Overt LC n=38/20/22/49 for blood-count measures, n=36/20/23/49 for albumin, and n=15/11/19/47 for CEA.

3. Unavailable covariates were smoking history, COPD history, BMI, recent infection history, treatment status at blood draw, flow-cytometry batch, and blood draw date.

4. PN pathology percentages use the applicable subgroup denominator. Overt LC histology and stage percentages use the Overt LC denominator.

**Figure 1:**
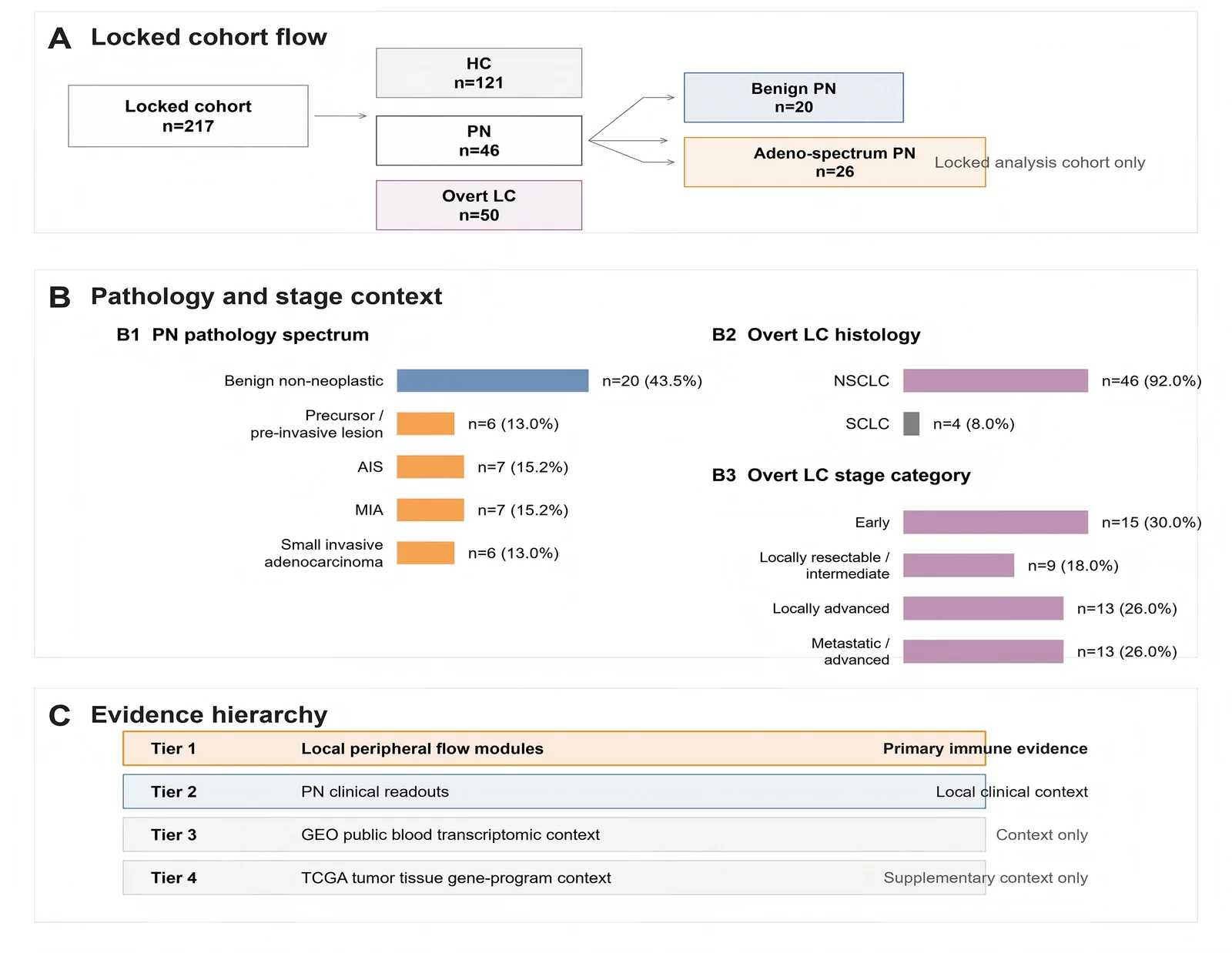
Study design, pathology context, and evidence hierarchy. **(A)** locked analysis cohort flow. The figure shows the locked analysis cohort only because screening and exclusion data are unavailable. **(B)** PN pathology spectrum and Overt LC histology/stage context. Adeno-spectrum PN refers to pulmonary nodules enrolled through the nodule pathway and pathologically classified within the adenocarcinoma spectrum or as small invasive adenocarcinoma presenting through the pulmonary-nodule clinical pathway. Overt LC refers to pathologically confirmed primary lung cancer managed through the lung-cancer clinical pathway. **(C)** evidence hierarchy. GEO and TCGA are contextual evidence only, not validation of the local flow-cytometry findings. TCGA represents tumor tissue context and is not peripheral immune validation. HC, healthy controls; PN, pulmonary nodule; Adeno-PN, Adeno-spectrum pulmonary nodule; Overt LC, overt lung cancer group; NSCLC, non-small cell lung cancer; SCLC, small cell lung cancer.

Pathology among PN included 20 benign non-neoplastic lesions and 26 Adeno-spectrum lesions: precursor or pre-invasive lesion (n=6), AIS (n=7), MIA (n=7), and small invasive adenocarcinoma presenting through the pulmonary-nodule clinical pathway (n=6). Overt LC included NSCLC (n=46) and SCLC (n=4); stage categories were early (n=15), locally resectable or intermediate (n=9), locally advanced (n=13), and metastatic or advanced (n=13) (**Figure 1**; **Table 1**).

### Clinical inflammatory and lymphocyte-axis differences within PN

**Figure 2:**
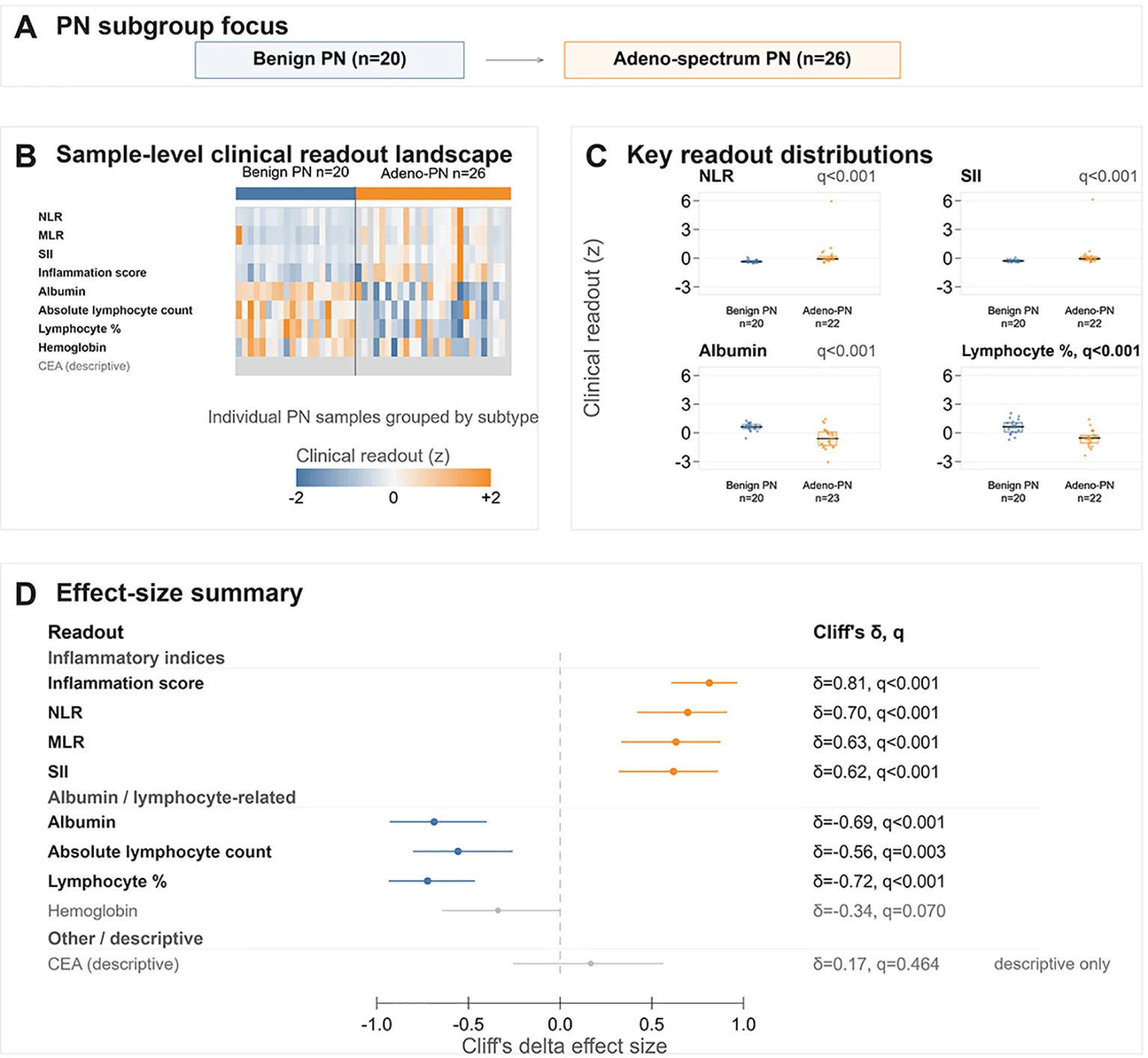
Clinical inflammatory and lymphocyte-axis differences within pulmonary nodules. **(A)** subgroup focus comparing Benign PN (n=20) with Adeno-spectrum PN (n=26). **(B)** sample-level clinical readout heatmap; columns represent individual PN samples grouped by subtype and rows represent clinical readouts. The heatmap uses precomputed z-scores calculated across PN samples separately for each clinical readout. Missing values are shown in light gray, and CEA is descriptive only. **(C)** complete-case distributions for NLR, SII, albumin, and lymphocyte percentage; each point represents one sample, and n varies across readouts because of missingness. **(D)** effect-size summary using precomputed Cliff’s δ and bootstrap 95% CI; the vertical dashed line indicates no directional difference, and positive Cliff’s δ indicates higher values in Adeno-spectrum PN. q values are from the locked BH-FDR-adjusted table. PN, pulmonary nodule; CI, confidence interval.

Compared with Benign PN, Adeno-spectrum PN showed a coherent pattern of higher inflammatory indices and lower lymphocyte-axis measures (**Figure 2**; **Table 2**). NLR (δ=0.70, q<0.001), MLR (δ=0.63, q<0.001), SII (δ=0.62, q<0.001), and the inflammation score (δ=0.81, q<0.001) were higher, whereas albumin (δ=−0.69, q<0.001), absolute lymphocyte count (δ=−0.56, q=0.003), and lymphocyte percentage (δ=−0.72, q<0.001) were lower. CEA remained descriptive only.

**Table 2:** Clinical readout comparisons within pulmonary nodules.

Characteristic	Benign PN, n	Adeno-spectrum PN, n	Benign PN, median [IQR]	Adeno-spectrum PN, median [IQR]	Cliff’s δ	95% CI for Cliff’s δ	P value	FDR q value	Direction
NLR	20	22	1.52 [1.20, 2.08]	2.81 [2.52, 4.08]	0.70	[0.42, 0.91]	<0.001	<0.001	Higher
MLR	20	22	0.21 [0.18, 0.23]	0.40 [0.26, 0.56]	0.63	[0.34, 0.87]	<0.001	<0.001	Higher
SII	20	22	451.00 [290.00, 629.00]	848 [637, 1,210]	0.62	[0.32, 0.86]	<0.001	<0.001	Higher
Inflammation score	20	22	-0.80 [-1.02, -0.53]	0.27 [-0.16, 0.82]	0.81	[0.61, 0.96]	<0.001	<0.001	Higher
Albumin, g/L	20	23	46.00 [45.00, 47.20]	39.70 [36.00, 43.20]	-0.69	[-0.93, -0.40]	<0.001	<0.001	Lower
Absolute lymphocyte count, ×10^9/L	20	22	1.91 [1.71, 2.40]	1.50 [1.05, 1.80]	-0.56	[-0.80, -0.26]	0.002	0.003	Lower
Lymphocyte, %	20	22	35.60 [29.60, 40.50]	22.90 [17.20, 26.00]	-0.72	[-0.93, -0.47]	<0.001	<0.001	Lower
Hemoglobin, g/L	20	22	140.00 [130.00, 150.00]	132.00 [115.00, 142.00]	-0.34	[-0.64, 0.00]	0.062	0.070	Lower
CEA, ng/mL	11	19	2.15 [1.34, 2.72]	2.53 [1.33, 4.16]	0.17	[-0.25, 0.56]	0.464	0.464	Descriptive only

1. Cliff’s δ is oriented as Adeno-spectrum PN relative to Benign PN; positive δ means higher values in Adeno-spectrum PN.

2. NLR = absolute neutrophil count / absolute lymphocyte count; MLR = absolute monocyte count / absolute lymphocyte count; SII = platelet count × absolute neutrophil count / absolute lymphocyte count.

3. Inflammation score = mean z(NLR, MLR, SII) − mean z(absolute lymphocyte count, albumin, hemoglobin), using population z-scoring over the locked analysis cohort and row-wise aggregation of available components.

4. CEA is descriptive only. P values, FDR q values, effect sizes, and confidence intervals were transferred unchanged from locked outputs.

### Descriptive peripheral immune-module structure

**Figure 3:**
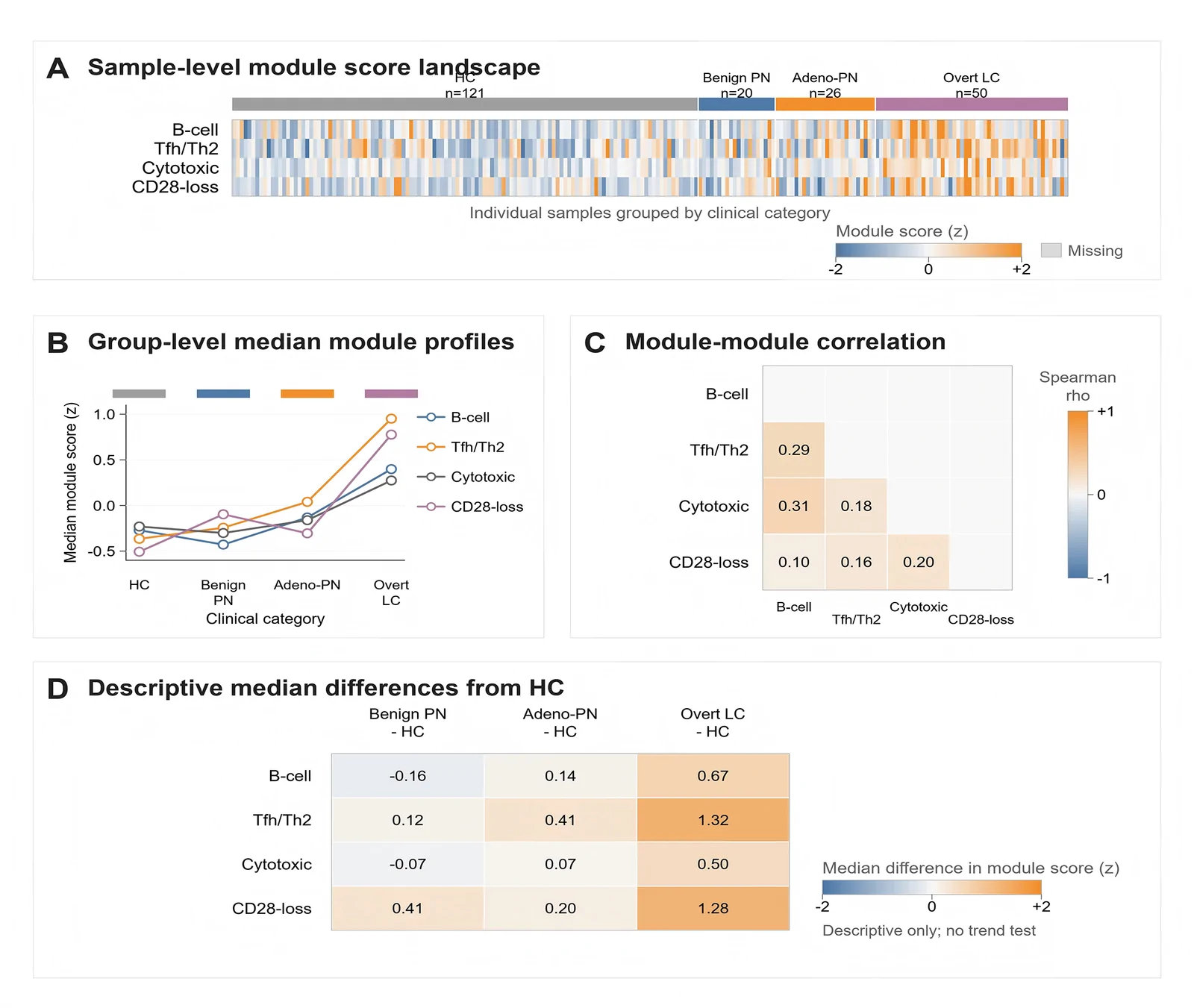
Global peripheral immune-module structure across HC, PN, and Overt LC. **(A)** sample-level z-scored module-score landscape; columns are individual samples grouped by clinical category. **(B)** group-level median module profiles. **(C)** module-module Spearman ρ matrix; q values are not shown because no locked q-value table was available. **(D)** descriptive median differences from HC. **Figure 3** is descriptive only: no ordered-trend test was performed and no longitudinal inference was made. HC, healthy controls; PN, pulmonary nodule; Adeno-PN, Adeno-spectrum pulmonary nodule; Overt LC, overt lung cancer group.

Across HC, Benign PN, Adeno-spectrum PN, and Overt LC, group-level median module profiles showed descriptive between-group differences (**Figure 3**). Overt LC had higher medians for several modules than HC and the PN subgroups, while pairwise module correlations were low to modest (Spearman ρ=0.10–0.31). **Figure 3** is descriptive only: no ordered-trend test was performed, and no longitudinal inference was made.

### Primary peripheral immune-module comparisons

**Figure 4:**
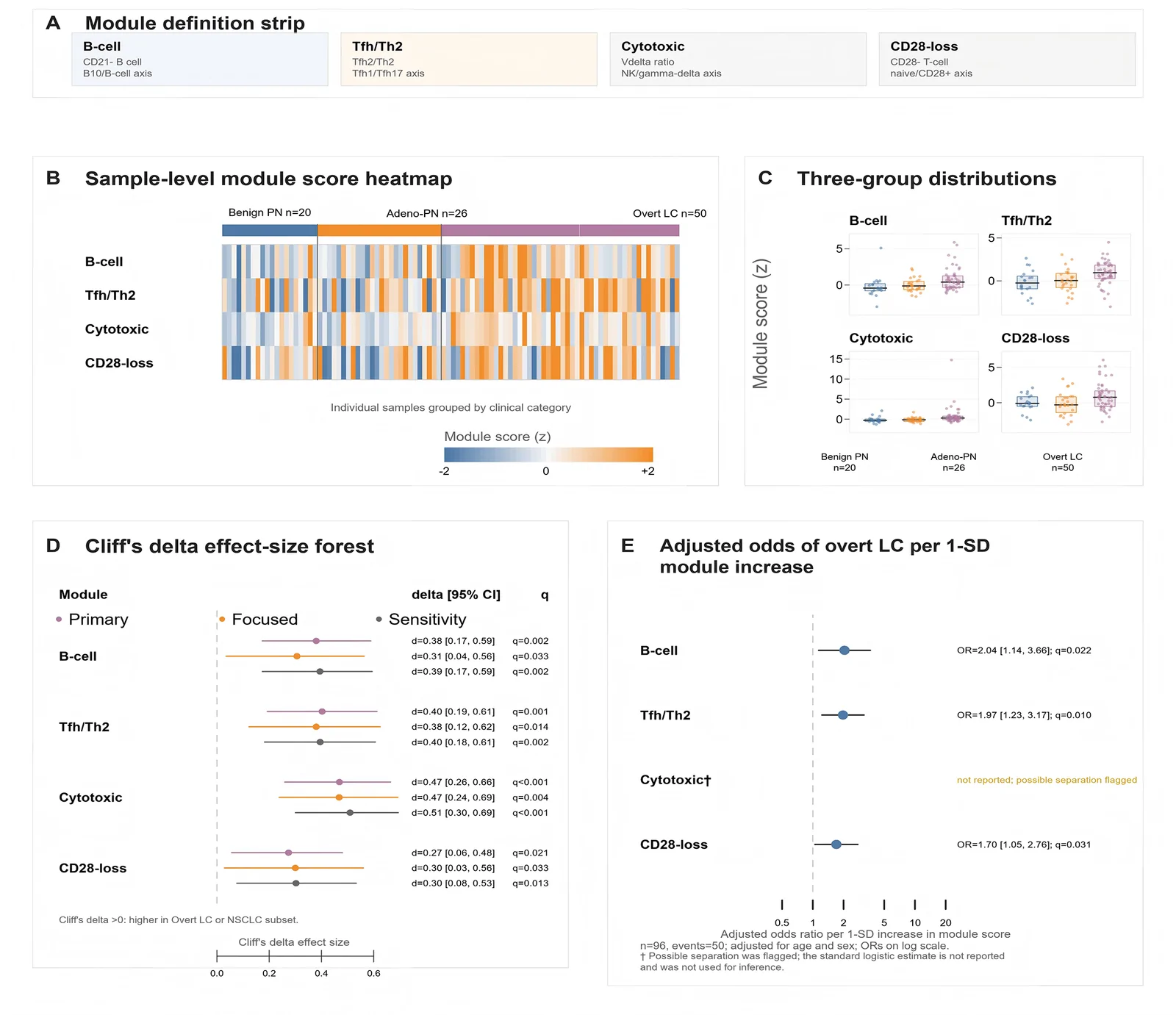
Code-defined peripheral flow modules are higher in overt LC than in pulmonary nodules. **(A)** compact definitions of the four code-defined peripheral flow modules. **(B)** sample-level module-score heatmap. **(C)** three-group module-score distributions. **(D)** Cliff’s δ effect-size forest for primary, focused, and sensitivity contrasts; positive δ indicates higher scores in Overt LC or the indicated cancer subset. **(E)** age- and sex-adjusted association estimates per 1-SD module increase. Cytotoxic attenuation is shown as “not reported; possible separation flagged.” Possible separation was flagged; the standard logistic estimate was not reported and was not used for inference. PN, pulmonary nodule; Adeno-PN, Adeno-spectrum pulmonary nodule; Overt LC, overt lung cancer group; NSCLC, non-small cell lung cancer; OR, odds ratio; CI, confidence interval; SD, standard deviation; q, Benjamini-Hochberg FDR-adjusted P value.

In the primary PN overall versus Overt LC comparison, all four module scores were higher in Overt LC: B-cell remodeling (δ=0.38, q=0.002), Tfh/Th2 skew (δ=0.40, q=0.001), Cytotoxic attenuation (δ=0.47, q<0.001), and CD28-loss module (δ=0.27, q=0.021) (**Figure 4**; **Table 3**). The directions were consistent in the focused comparison and sensitivity analyses. Sensitivity support was strongest for Cytotoxic attenuation and Tfh/Th2 skew, whereas B-cell remodeling and CD28-loss module had weaker support in selected restricted comparisons (**Supplementary Tables 2**, **S3**).

**Table 3:** Primary peripheral immune-module comparisons.

Module score	PN overall, median [IQR]	Overt LC, median [IQR]	Cliff’s δ	95% CI for Cliff’s δ	P value	FDR q value
B-cell remodeling	-0.32 [-0.74, 0.39]	0.40 [-0.33, 1.26]	0.38	[0.17, 0.59]	0.001	0.002
Tfh/Th2 skew	0.00 [-0.87, 0.78]	0.95 [0.27, 1.84]	0.40	[0.19, 0.61]	<0.001	0.001
Cytotoxic attenuation	-0.23 [-0.57, 0.14]	0.27 [-0.10, 0.74]	0.47	[0.26, 0.66]	<0.001	<0.001
CD28-loss module	-0.22 [-0.59, 0.87]	0.78 [-0.54, 1.68]	0.27	[0.06, 0.48]	0.021	0.021

1. Primary comparison: PN overall (comparator, n=46) versus Overt LC (n=50); group values are median [IQR].

2. Cliff’s δ is oriented as Overt LC relative to PN; positive δ means higher module scores in Overt LC.

3. Possible separation was flagged for Cytotoxic attenuation; the standard logistic estimate was not reported and was not used for inference.

4. Focused and sensitivity comparisons are reported separately in **Supplementary Tables S2**, **S3**.

Age- and sex-adjusted models were supportive diagnostics. For Cytotoxic attenuation, the standard logistic estimate was not reported because possible separation was flagged; inference used the nonparametric effect-size comparison (**Figure 4E**; **Supplementary Table 4**).

## Discussion

This study provides an exploratory peripheral immunophenotyping framework across benign PNs), adenocarcinoma-spectrum PN, and overt LC. Two complementary peripheral blood layers were observed: clinical inflammatory and lymphocyte-related differences within PN, and higher flow-cytometry module scores in overt LC than PN. These findings represent cross-sectional group associations and do not establish temporal progression or mechanistic immune states.

The PN clinical-readout findings indicate that adenocarcinoma-spectrum PN differ edfrom benign PN in routine systemic readouts. Higher NLR, MLR, SII, and inflammation score, together with lower albumin and lymphocyte measures, describe a coherent inflammatory, nutritional, and lymphocyte-related pattern. Prior studies have associated NLR and SII with outcomes in established cancers (6, 8). However, in the present PN cohort these indices should be interpreted as nonspecific systemic readouts rather than evidence of a tumor-promoting mechanism or an early causal event.

PNs are clinically heterogeneous, and their interpretation depends on radiological characteristics, underlying lung disease, and longitudinal context. Coexisting interstitial lung disease can alter nodule assessment and management (37). and a recent longitudinal multimodal study in fibrosing interstitial lung disease further illustrated the importance of integrating imaging and clinical context (38). Multimodal longitudinal approaches may therefore complement peripheral biological readouts (39). The present study did not systematically analyze radiological morphology or serial imaging.

The flow-cytometry findings extend this clinical-readout layer by summarizing multiple marker-defined peripheral immune phenotypes into four transparent code-defined modules. Overt LC showed higher B-cell remodeling, Tfh/Th2 skew, cytotoxic attenuation, and CD28-loss module scores than PN. These modules should be interpreted as compact summaries of related immune phenotypes rather than as mechanistic entities. The elevation of the B-cell remodeling module in overt LC is consistent with the emerging understanding of B-cell involvement in tertiary lymphoid structures (TLS) within tumors. Sarvaria et al. noted that while B cells can regulate anti-tumor immunity, specific subsets may also contribute to immune evasion (21, 27–29). but these literature associations do not establish that the peripheral modules measured here reflect tumor tertiary lymphoid structures, tissue immune states, or specific mechanisms. The present results therefore support between-group differences in analytical module scores, not mechanistic equivalence.

Among the modules, cytotoxic attenuation and Tfh/Th2 skew showed the most consistent statistical support across primary, focused, and sensitivity comparisons. Previous studies have described CD8+ T-cell subset shifts and associations of CD28 expression with clinical outcomes in lung cancer (15–17). In the present study, however, the marker-defined cytotoxic attenuation and CD28-loss modules did not directly measure effector function, exhaustion, immunosenescence, or treatment responsiveness. Their functional and therapeutic implications remain to be established. Nonparametric effect sizes and sensitivity consistency were therefore treated as the primary evidence, with adjusted models used only as supportive diagnostics.

The module analysis also included NK-cell and gamma-delta T-cell phenotypes. These cell types have established roles in cancer immunity and immunotherapy research (22, 23, 26). providing context for the selected marker-defined components. The current cross-sectional peripheral blood data do not determine whether the observed module differences reflect antitumor activity, immune suppression, or treatment-relevant function.

The novelty of this work lies in the combination of clinical pathway stratification, local whole-blood multiparameter flow cytometry, transparent immune-module construction, and a structured evidence hierarchy. Public transcriptomic and tissue resources were retained only as supplementary context for compartment-level interpretation; they were not used as external validation of the local peripheral flow-cytometry findings.

Several limitations should be considered. First, the study was retrospective and cross-sectional, and cannot establish temporal ordering, causality, or longitudinal immune change. Second, the PN subgroup comparison was modest in size, and complete-case availability varies across readouts. Third, smoking history, comorbidity burden, medication use, infection history, and detailed radiological variables were unavailable or incompletely captured, leaving substantial potential for residual confounding. Fourth, adjusted logistic models were constrained by sample size and possible separation for some predictors. Although several sensitivity analyses retained the direction of associations, these analyses do not eliminate confounding or establish pathological specificity.

Prospective studies with larger cohorts, standardized processing, comprehensive clinical and radiological covariates, and independent replication are needed to assess reproducibility. Longitudinal and paired tissue-peripheral designs may further clarify temporal relationships and biological interpretation, but causal or clinically actionable conclusions cannot be drawn from the present data.

## Conclusion

In summary, adenocarcinoma-spectrum PN was associated with a clinical inflammatory and lymphocyte-related pattern relative to benign PN, while overt LC showed higher code -defined flow-cytometry module scores than PN. These cross-sectional findings are association-based and hypothesis-generating. Prospective studies with richer covariate capture, standardized flow-cytometry quality control, independent replication, and paired tissue-peripheral sampling are required before functional or clinical interpretation.

## Data Availability

The original contributions presented in the study are included in the article/**Supplementary Material**. Further inquiries can be directed to the corresponding author/s.

## Corection note

A correction has been made to this article. Details can be found at: 10.3389/fimmu.2026.2013441.
