# Correction: Clinical inflammatory shifts and peripheral flow-cytometry immune modules across benign pulmonary nodules, adenocarcinoma-spectrum nodules, and overt lung cancer

**DOI:** 10.3389/fimmu.2026.2013441

**Published:** 2026-10-20

**Authors:** Yunwei Rao, Qianyue Gong, Jiawei Li, Nan Liu, Hongmei Miao, Jinyue Huang, Shuiming Xu

**Affiliations:** 1 Department of Respiratory and Critical Care Medicine, First Affiliated Hospital of Gannan Medical University, Ganzhou, Jiangxi, China; 2 Medical School, Shaoguan University, Guangzhou, Guangdong, China; 3 Institute of Biomedical Translational Research, Jinan University, Guangzhou, Guangdong, China; 4 School of Public Health and Health Management, Gannan Medical University, Ganzhou, Jiangxi, China; 5 Guangdong Provincial People’s Hospital Ganzhou Hospital (Ganzhou Municipal Hospital), Ganzhou, Jiangxi, China; 6 School of Clinical Medicine, Nanchang Medical College, Nanchang, Jiangxi, China

**Keywords:** clinical inﬂammation, exploratory cohort, ﬂow cytometry, immune module, lung cancer, lymphocyte axis, peripheral immunity, pulmonary nodule

The affiliation assigned to co-author Nan Liu was erroneously recorded in the published article. The correct affiliations for Nan Liu are “Guangdong Provincial People’s Hospital Ganzhou Hospital (Ganzhou Municipal Hospital) and School of Public Health and Health Management, Gannan Medical University,” rather than what currently appears.

The original version of this article has been updated.

