# Supplementary material for: Clinical inflammatory shifts and peripheral flow-cytometry immune modules across benign pulmonary nodules, adenocarcinoma-spectrum nodules, and overt lung cancer
Source: Front Immunol. 2026 Sep 1;17:1920402. doi: 10.3389/fimmu.2026.1920402 (PMC13574593; doi:10.3389/fimmu.2026.1920402)
Supplement: Supplementary file 1 [file DataSheet1.docx]

Supplementary Material

# Supplementary Tables

| **Supplementary Table S1.**  **Focused module comparisons** | | | | | | | | | |
| --- | --- | --- | --- | --- | --- | --- | --- | --- | --- |
| **Comparison** | **Module score** | **Adeno-PN n** | **Overt LC n** | **Adeno-PN median [IQR]** | **Overt LC median [IQR]** | **Cliff’s δ** | **95% CI for Cliff’s δ** | **P value** | **FDR q value** |
| Adeno-PN vs Overt LC | B-cell remodeling | 26 | 50 | -0.13 [-0.64, 0.49] | 0.40 [-0.33, 1.26] | 0.31 | [0.04, 0.56] | 0.030 | 0.033 |
|  | Tfh/Th2 skew | 26 | 50 | 0.04 [-0.78, 0.86] | 0.95 [0.27, 1.84] | 0.38 | [0.12, 0.62] | 0.007 | 0.014 |
|  | Cytotoxic attenuation | 26 | 50 | -0.16 [-0.57, 0.18] | 0.27 [-0.10, 0.74] | 0.47 | [0.24, 0.69] | <0.001 | 0.004 |
|  | CD28-loss module | 26 | 50 | -0.30 [-1.40, 0.85] | 0.78 [-0.54, 1.68] | 0.30 | [0.03, 0.56] | 0.033 | 0.033 |
| Notes: | | | | | | | | | |
| 1. Focused comparison is Adeno-PN vs Overt LC; Adeno-PN retains small invasive adenocarcinoma when presenting through the pulmonary-nodule clinical pathway. | | | | | | | | | |
| 2. Cliff's δ direction is Overt LC relative to Adeno-PN; positive δ indicates higher module score in Overt LC, defined as pathologically confirmed primary lung cancer managed through the lung-cancer clinical pathway. | | | | | | | | | |
| 3. Nonparametric effect-size statistics were taken from analysis-locked outputs. | | | | | | | | | |

| **Supplementary Table S2.**  **Sensitivity analyses of module-score comparisons** | | | | | | | |
| --- | --- | --- | --- | --- | --- | --- | --- |
| **Sensitivity analysis** | **Module score** | **Group 1 n** | **Group 2 n** | **Cliff’s δ** | **95% CI for Cliff’s δ** | **P value** | **FDR q value** |
| PN vs NSCLC only | B-cell remodeling | 46 | 46 | 0.39 | [0.17, 0.59] | 0.001 | 0.002 |
| PN vs NSCLC only | Tfh/Th2 skew | 46 | 46 | 0.40 | [0.18, 0.61] | 0.001 | 0.002 |
| PN vs NSCLC only | Cytotoxic attenuation | 46 | 46 | 0.51 | [0.30, 0.69] | <0.001 | <0.001 |
| PN vs NSCLC only | CD28-loss module | 46 | 46 | 0.30 | [0.08, 0.53] | 0.013 | 0.013 |
| PN vs LC excluding stage IV | B-cell remodeling | 46 | 37 | 0.35 | Not estimated | 0.006 | 0.008 |
| PN vs LC excluding stage IV | Tfh/Th2 skew | 46 | 37 | 0.36 | Not estimated | 0.006 | 0.008 |
| PN vs LC excluding stage IV | Cytotoxic attenuation | 46 | 37 | 0.47 | Not estimated | <0.001 | <0.001 |
| PN vs LC excluding stage IV | CD28-loss module | 46 | 37 | 0.25 | Not estimated | 0.056 | 0.056 |
| Adeno-PN vs LUAD only | B-cell remodeling | 26 | 28 | 0.23 | Not estimated | 0.148 | 0.148 |
| Adeno-PN vs LUAD only | Tfh/Th2 skew | 26 | 28 | 0.36 | Not estimated | 0.024 | 0.048 |
| Adeno-PN vs LUAD only | Cytotoxic attenuation | 26 | 28 | 0.57 | Not estimated | <0.001 | 0.001 |
| Adeno-PN vs LUAD only | CD28-loss module | 26 | 28 | 0.25 | Not estimated | 0.113 | 0.148 |
| Notes: | | | | | | | |
| 1. Sensitivity analyses preserve the direction of the main module findings, with strongest statistical support for Cytotoxic attenuation and Tfh/Th2 skew. | | | | | | | |
| 2. B-cell remodeling and CD28-loss module are supportive but weaker in selected restricted comparisons. | | | | | | | |
| 3. Bootstrap confidence intervals were unavailable in the analysis-locked output for selected sensitivity contrasts; these CIs were not estimated in this formatting step. | | | | | | | |

| **Supplementary Table S3.**  **Adjusted logistic model diagnostics** | | | | | | | | | |
| --- | --- | --- | --- | --- | --- | --- | --- | --- | --- |
| **Model family** | **Outcome** | **Predictor** | **n** | **Events** | **OR per 1-SD** | **95% CI** | **P value** | **FDR q value** | **Covariates** |
| Peripheral flow module | Overt LC vs PN overall | B-cell remodeling | 96 | 50 | 2.04 | [1.14, 3.66] | 0.016 | 0.022 | Age + sex |
|  |  | Tfh/Th2 skew | 96 | 50 | 1.97 | [1.23, 3.17] | 0.005 | 0.010 | Age + sex |
|  |  | Cytotoxic attenuation | 96 | 50 | Unstable / not reported | Unstable / not reported | 0.005 | 0.010 | Age + sex |
|  |  | CD28-loss module | 96 | 50 | 1.70 | [1.05, 2.76] | 0.031 | 0.031 | Age + sex |
| Clinical readout |  | NLR | 91 | 49 | 1.34 | [0.77, 2.34] | 0.306 | 0.378 | Age + sex |
|  |  | MLR | 91 | 49 | 1.20 | [0.58, 2.48] | 0.624 | 0.624 | Age + sex |
|  |  | SII | 91 | 49 | 1.32 | [0.75, 2.30] | 0.336 | 0.378 | Age + sex |
|  |  | Inflammation score | 91 | 49 | 1.82 | [1.01, 3.29] | 0.046 | 0.103 | Age + sex |
|  |  | Albumin | 92 | 49 | 0.58 | [0.35, 0.95] | 0.030 | 0.090 | Age + sex |
|  |  | Hemoglobin | 91 | 49 | 0.62 | [0.38, 1.01] | 0.057 | 0.103 | Age + sex |
|  |  | Absolute lymphocyte count | 91 | 49 | 0.67 | [0.43, 1.06] | 0.084 | 0.127 | Age + sex |
|  |  | Lymphocyte % | 91 | 49 | 0.57 | [0.35, 0.94] | 0.028 | 0.090 | Age + sex |
|  |  | CEA (descriptive) | 77 | 47 | Unstable / not reported | Unstable / not reported | 0.028 | 0.090 | Age + sex |
|  | Adeno-spectrum PN vs Benign PN | NLR | 42 | 22 | Unstable / not reported | Unstable / not reported | 0.007 | 0.013 | Age + sex |
|  |  | MLR | 42 | 22 | 2.17 | [0.84, 5.61] | 0.111 | 0.125 | Age + sex |
|  |  | SII | 42 | 22 | Unstable / not reported | Unstable / not reported | 0.006 | 0.013 | Age + sex |
|  |  | Inflammation score | 42 | 22 | Unstable / not reported | Unstable / not reported | 0.002 | 0.011 | Age + sex |
|  |  | Albumin | 43 | 23 | 0.18 | [0.05, 0.58] | 0.004 | 0.013 | Age + sex |
|  |  | Hemoglobin | 42 | 22 | 0.30 | [0.10, 0.89] | 0.030 | 0.038 | Age + sex |
|  |  | Absolute lymphocyte count | 42 | 22 | 0.26 | [0.08, 0.78] | 0.016 | 0.025 | Age + sex |
|  |  | Lymphocyte % | 42 | 22 | 0.16 | [0.05, 0.52] | 0.002 | 0.011 | Age + sex |
|  |  | CEA (descriptive) | 30 | 19 | 1.70 | [0.59, 4.88] | 0.323 | 0.323 | Age + sex |
| Notes: | | | | | | | | | |
| 1. Supplementary adjusted logistic model status only; estimates were not reported when model status suggested quasi-complete separation or unstable effect estimation. | | | | | | | | | |
| 2. Possible separation was flagged for Cytotoxic attenuation; the standard logistic estimate was not used for inference and is not reported as an interpretable adjusted OR. | | | | | | | | | |
| 3. Bias-reduced/Firth sensitivity was unavailable in analysis-locked outputs; unstable standard estimates were not used for primary claims. | | | | | | | | | |

| **Supplementary Table S4.**  **Flow-cytometry phenotype definitions** | | | |
| --- | --- | --- | --- |
| **Panel** | **Publication label** | **Marker definition** | **Denominator / unit** |
| Panel 1 T-cell overview | T cell | CD3+ | % of lymphocyte |
|  | T cell count | — | cells/μL |
|  | CD4+ T cells | CD3+CD4+ | % of T cells |
|  | CD8+ T cells | CD3+CD8+ | % of T cells |
|  | (CD3+CD4+) / (CD3+CD8+) ratio | (CD3+CD4+)/(CD3+CD8+) | — |
|  | Double negative T cell | CD3+CD4-CD8- | % of T cells |
|  | Naive CD4+ T cell | CD3+CD4+CD45RA+CCR7+ | % of CD4+ T cells |
|  | Terminally differentiated CD4+ T cell | CD3+CD4+CD45RA+CCR7- | % of CD4+ T cells |
|  | Central memory CD4+ T cell | CD3+CD4+CD45RA-CCR7+ | % of CD4+ T cells |
|  | Effector memory CD4+ T cell | CD3+CD4+CD45RA-CCR7- | % of CD4+ T cells |
|  | CD4+CD28- T cells | CD3+CD4+CD28- | % of CD4+ T cells |
|  | CD4+CD28+ T cells | CD3+CD4+CD28+ | % of CD4+ T cells |
|  | CD4+CD25+CD127low/− T cells | CD3+CD4+CD25+CD127low/- | % of CD3+T cells |
|  | Naive CD8+ T cell | CD3+CD8+CCR7+CD45RA+ | % of CD8+ T cells |
|  | Terminally differentiated CD8+ T cell | CD3+CD8+CCR7-CD45RA+ | % of CD8+ T cells |
|  | Central memory CD8+ T cell | CD3+CD8+CCR7+CD45RA- | % of CD8+ T cells |
|  | Effector memory CD8+ T cell | CD3+CD8+CCR7-CD45RA- | % of CD8+ T cells |
|  | CD8+CCR7−CD45RA−CD127+ T cells | CD3+CD8+CCR7-CD45RA-CD127+ | % of Effective memory CD8+ T cells |
|  | CD8+CCR7−CD45RA−CD127− T cells | CD3+CD8+CCR7-CD45RA-CD127- | % of Effective memory CD8+ T cells |
|  | CD8+CCR7−CD45RA+CD127+ T cells | CD3+CD8+CCR7-CD45RA+CD127+ | % of Terminally differentiated CD8+ T cells |
|  | CD8+CCR7−CD45RA+CD127− T cells | CD3+CD8+CCR7-CD45RA+CD127- | % of Terminally differentiated CD8+ T cells |
| Panel 2 T-helper/T-cell subset panel | Tfh | CD3+CD4+CXCR5+ | %of CD4+ T cells |
|  | Tfh1 | CD3+CD4+CXCR5+CXCR3+CCR4- | %of Tfh cells |
|  | Tfh2 | CD3+CD4+CXCR5+CXCR3-CCR4+ | %of Tfh cells |
|  | Tfh17 | CD3+CD4+CXCR5+CXCR3-CCR4-CCR6+ | %of Tfh cells |
|  | Th1 | CD3+CD4+CXCR5-CXCR3+CCR4- | %of CD4+ T cells |
|  | Th2 | CD3+CD4+CXCR5-CXCR3-CCR4+ | %of CD4+ T cells |
|  | Th17 | CD3+CD4+CXCR5-CXCR3-CCR4-CCR6+ | %of CD4+ T cells |
|  | Th1 / Th2 ratio | Th1/Th2 | — |
|  | Th17 / Th2 ratio | Th17/Th2 | — |
|  | (Th1+Th17) / Th2 ratio | (Th1+Th17)/Th2 | — |
|  | Tc1 | CD3+CD8+CXCR5-CXCR3+CCR4- | %of CD8+ T cells |
|  | Tc2 | CD3+CD8+CXCR5-CXCR3-CCR4+ | %of CD8+ T cells |
|  | Tc17 | CD3+CD8+CXCR5-CXCR3-CCR4-CCR6+ | %of CD8+ T cells |
|  | Peripheral helper T cell | CD3+CD4+CXCR5-PD-1+ | of CD4+ T cells |
|  | CD4+CXCR5+PD-1+ T cells | CD3+CD4+CXCR5+PD-1+ | of CD4+ T cells |
| Panel 3 CD8 T-cell subset panel | CD8+PD-1+ T cells | CD3+CD8+PD-1+ | % of CD8+ T cells |
|  | CD8+CD28- T cells | CD3+CD8+CD28- | % of CD8+ T cells |
|  | CD8+CD28+ T cells | CD3+CD8+CD28+ | % of CD8+ T cells |
|  | Total memory CD8+ T cell | CD3+CD8+HLADR+ | % of CD8+ T cells |
|  | Homing memory CD8+ T cell | CD3+CD8+HLADR+CD38+ | % of CD3+CD8+HLADR+ |
|  | CD8+CD28-CD57+ T cells | CD3+CD8+CD28-CD57+ | % of CD8+ T cells |
| Panel 4 NK-cell panel | T-NK cell | CD3+CD56+ | % of lymphocyte |
|  | NK cell | CD3-CD56+ | % of lymphocyte |
|  | NK cell count | — | cells/μL |
|  | CD3−CD56+bright NK cells | CD3-CD56+bright | % of NK cells |
|  | CD3−CD56+dim NK cells | CD3-CD56+dim | % of NK cells |
|  | (CD3−CD56+bright) / (CD3−CD56+dim) ratio | (CD3-CD56+bright)/(CD3-CD56+dim) | — |
|  | CD3−CD56+CD94+KIR− NK cells | CD3-CD56+CD94+KIR- | % of NK cells |
|  | CD3−CD56+CD94−KIR+ NK cells | CD3-CD56+CD94-KIR+ | % of NK cells |
|  | CD3−CD56+NKG2D+ NK cells | CD3-CD56+NKG2D+ | % of NK cells |
|  | CD3−CD56+NKp30+ NK cells | CD3-CD56+NKP30+ | % of NK cells |
|  | CD3−CD56+NKp46+ NK cells | CD3-CD56+NKP46+ | % of NK cells |
| Panel 5 γδ T-cell panel | γδ T cell | CD3+γδT+ | % of T cells |
|  | Vδ1+ T cell | CD3+γδT+Vδ1+ | % of γδT cells |
|  | Vδ2+ T cell | CD3+γδT+Vδ2+ | %of γδT cells |
|  | Vδ1+ T cell/Vδ2+ T cell ratio | — | — |
|  | Vδ2+NKG2D+ γδ T cells | CD3+γδT+Vδ2+NKG2D+ | % of Vδ2+γδT cells |
|  | Vδ2+PD-1+ γδ T cells | CD3+γδT+Vδ2+PD1+ | % of Vδ2+ γδT cells |
|  | Vδ2+NKp30+ γδ T cells | CD3+γδT+Vδ2+NKP30+ | % of Vδ2+ γδT cells |
|  | Vδ2+NKp46+ γδ T cells | CD3+γδT+Vδ2+NKP46+ | % of Vδ2+ γδT cells |
|  | Vδ1+NKG2D+ γδ T cells | CD3+γδT+Vδ1+NKG2D+ | % of Vδ1+γδT cells |
|  | Vδ1+PD-1+ γδ T cells | CD3+γδT+Vδ1+PD-1+ | % of Vδ1+γδT cells |
|  | Vδ1+NKp30+ γδ T cells | CD3+γδT+Vδ1+NKP30+ | % of Vδ1+γδT cells |
|  | Vδ1+NKp46+ γδ T cells | CD3+γδT+Vδ1+NKP46+ | % of Vδ1+γδT cells |
| Panel 6 B-cell panel | B cell | CD3-CD19+ | % of lymphocyte |
|  | B cell count | — | cells/μL |
|  | Naive B cell | CD3-CD19+CD27-IgD+ | % of B cells |
|  | MZ B cell | CD3-CD19+CD27+IgD+ | % of B cells |
|  | CD21- B cell | CD3-CD19+CD38-CD21- | % of B cells |
|  | Pre-naive B cell | CD3-CD19+IgM-IgD-CD27-CD38+ | % of B cells |
|  | Plasmablasts | CD3-CD19+IgD-IgM-CD27+CD38+ | % of B cells |
|  | Class-switched B cells | CD3-CD19+IgD-IgM-CD27+CD38- | % of B cells |
|  | B10 cell | CD3-CD19+IgD+IgM+CD27+CD24+ | % of B cells |
|  | Memory B cell | CD3-CD19+CD27+CD38- | % of B cells |
|  | Non-switched B cells | CD3-CD19+IgM+CD27+CD38- | % of B cells |
|  | CD3−CD19+IgD+IgM+CD24+CD38+ B cells | CD3-CD19+IgD+IgM+CD24+CD38+ | % of B cells |
|  | Transitional B cells | CD3-CD19+CD27-CD38+IgM+CD24+ | % of B cells |

| **Supplementary Table S5.**  **Flow-cytometry antibody panel** | | | | | |
| --- | --- | --- | --- | --- | --- |
| **Panel / tube** | **Marker** | **Fluorochrome** | **Clone** | **Supplier** | **Catalog number** |
| T | CD3 | PerCP-Cy5.5 | UCHT1 | BD Biosciences / BD Pharmingen | 560835 |
| T | CD4 | APC-H7 | RPA-T4 | BD Biosciences / BD Pharmingen | 560158 |
| T | CD8 | BV510 | SK1 | BD Biosciences / BD Horizon | 563919 |
| T | CD28 | PE-Cy7 | CD28.2 | BD Biosciences / BD Pharmingen | 560684 |
| T | CD127 | BV421 | hIL-7R-M21 | BD Biosciences / BD Horizon | 562436 |
| T | CCR7（CD197） | AF647 | 150503 | BD Biosciences / BD Pharmingen | 560816 |
| T | CD25 | PE | M-A251 | BD Biosciences / BD Pharmingen | 555432 |
| T | CD45RA | FITC | HI100 | BD Biosciences / BD Pharmingen | 555488 |
| CD8T | CD3 | PerCP-Cy5.5 | UCHT1 | BD Biosciences / BD Pharmingen | 560835 |
| CD8T | CD28 | PE-Cy7 | CD28.2 | BD Biosciences / BD Pharmingen | 560684 |
| CD8T | CD57 | BV421 | QA17A04 | BioLegend | 393326 |
| CD8T | CD38 | BV510 | HIT2 | BD Biosciences / BD Horizon | 563251 |
| CD8T | PD-1(CD279) | AF647 | EH12.1 | BD Biosciences / BD Pharmingen | 560838 |
| CD8T | CD8 | FITC | HIT8a | BD Biosciences / BD Pharmingen | 555634 |
| CD8T | HLA-DR | PE | G46-6 | BD Biosciences / BD Pharmingen | 555812 |
| NK | CD3 | APC-H7 | SK7 | BD Biosciences / BD Pharmingen | 560176 |
| NK | CD56 | APC | B159 | BD Biosciences / BD Pharmingen | 555518 |
| NK | CD94 | PerCP-Cy5.5 | HP-3D9 | BD Biosciences / BD Pharmingen | 562361 |
| NK | NKG2D(CD314) | PE-Cy7 | 1D11 | BD Biosciences / BD Pharmingen | 562365 |
| NK | NKP30(CD337) | BV421 | p30-15 | BD Biosciences / BD Horizon | 563385 |
| NK | NKP46(CD335) | BV510 | 9E2/NKp46 | BD Biosciences / BD Horizon | 564064 |
| NK | NKB1 | PE | DX9 | BD Biosciences / BD Pharmingen | 555967 |
| GDT | CD3 | APC-H7 | SK7 | BD Biosciences / BD Pharmingen | 560176 |
| GDT | TCR gd | BV421 | 11F2 | BD Biosciences / BD OptiBuild | 744870 |
| GDT | PD-1(CD279) | BB515 | EH12.1 | BD Biosciences / BD Horizon | 564494 |
| GDT | NKP46(CD335) | BV510 | 9E2/NKp46 | BD Biosciences / BD Horizon | 564064 |
| GDT | NKP30(CD337) | AF647 | p30-15 | BD Biosciences / BD Pharmingen | 558408 |
| GDT | NKG2D(CD314) | PE-Cy7 | 1D11 | BD Biosciences / BD Pharmingen | 562365 |
| GDT | TCR Vd2 | PE | B6 | BD Biosciences / BD Pharmingen | 555739 |
| GDT | TCR Vd1 | percp-vio700 | REA173 | Miltenyi Biotec | 130-120-441 |
| B | CD45 | APC-H7 | 2D1 | BD Biosciences / BD Pharmingen | 560178 |
| B | CD19 | PerCP-Cy5.5 | HIB19 | BD Biosciences / BD Pharmingen | 561295 |
| B | CD27 | BV421 | M-T271 | BD Biosciences / BD Horizon | 562513 |
| B | IgD | BB515 | IA6-2 | BD Biosciences / BD Horizon | 565243 |
| B | IgM | BV510 | G20-127 | BD Biosciences / BD Horizon | 563113 |
| B | CD38 | APC | HIT2 | BD Biosciences / BD Pharmingen | 555462 |
| B | CD24 | PE | ML5 | BD Biosciences / BD Pharmingen | 555428 |
| B | CD21 | PE-Cy7 | B-ly4 | BD Biosciences / BD Pharmingen | 561374 |
| Th | CD3 | APC-H7 | SK7 | BD Biosciences / BD Pharmingen | 560176 |
| Th | CD4 | PE-Cy7 | SK3 | BD Biosciences / BD Pharmingen | 557852 |
| Th | CD8 | PerCP-Cy5.5 | SK1 | BD Biosciences / BD Pharmingen | 565310 |
| Th | CD183(CXCR3) | AF488 | 1C6/CXCR3 | BD Biosciences / BD Pharmingen | 558047 |
| Th | CD196(CCR6) | BV510 | 11A9 | BD Biosciences / BD Horizon | 563241 |
| Th | CD185(CXCR5) | AF647 | RF8B2 | BD Biosciences / BD Pharmingen | 558113 |
| Th | CD194 | BV421 | 1G1 | BD Biosciences / BD Horizon | 562579 |
| Th | PD-1(CD279) | PE | MIH4 | BD Biosciences / BD Pharmingen | 557946 |

| **Supplementary Table S6.**  **Module score definitions** | | | | | |
| --- | --- | --- | --- | --- | --- |
| **Module score** | **Component phenotype** | **Marker definition / component label** | **Direction in score** | **Transformation** | **Aggregation method** |
| B-cell remodeling | CD21- B cell | CD3-CD19+CD38-CD21- | +1 positive term | population z-score for each component variable before aggregation | mean(z(positive_terms)) - mean(z(negative_terms)) |
|  | B10 cell | CD3-CD19+IgD+IgM+CD27+CD24+ | -1 negative term |  |  |
|  | B cell | CD3-CD19+ | -1 negative term |  |  |
|  | MZ B cell | CD3-CD19+CD27+IgD+ | -1 negative term |  |  |
|  | Non-switched B cells | CD3-CD19+IgM+CD27+CD38- | -1 negative term |  |  |
|  | Transitional B cells | CD3-CD19+CD27-CD38+IgM+CD24+ | -1 negative term |  |  |
|  | Immature regulatory B cell | CD3-CD19+IgD+IgM+CD24+CD38+ | -1 negative term |  |  |
| Tfh/Th2 skew | Tfh2 | CD3+CD4+CXCR5+CXCR3-CCR4+ | +1 positive term |  |  |
|  | Th2 | CD3+CD4+CXCR5-CXCR3-CCR4+ | +1 positive term |  |  |
|  | Tfh1 | CD3+CD4+CXCR5+CXCR3+CCR4- | -1 negative term |  |  |
|  | Tfh17 | CD3+CD4+CXCR5+CXCR3-CCR4-CCR6+ | -1 negative term |  |  |
|  | Th17 | CD3+CD4+CXCR5-CXCR3-CCR4-CCR6+ | -1 negative term |  |  |
|  | Th17/Th2 ratio | Th17/Th2 | -1 negative term |  |  |
| Cytotoxic attenuation | Vδ1+ T cell/Vδ2+ T cell ratio | Author-confirmed dimensionless ratio: (% Vδ1⁺ γδ T cells within γδ T cells) / (% Vδ2⁺ γδ T cells within γδ T cells). | +1 positive term |  |  |
|  | Vδ2+ T cell | CD3+γδT+Vδ2+ | -1 negative term |  |  |
|  | γδ T cell | CD3+γδT+ | -1 negative term |  |  |
|  | NKG2D+ NK cells | CD3-CD56+NKG2D+ | -1 negative term |  |  |
|  | NKG2D+ Vδ2 γδ T cells | CD3+γδT+Vδ2+NKG2D+ | -1 negative term |  |  |
| CD28-loss module | CD4+CD28− T cells | CD3+CD4+CD28- | +1 positive term |  |  |
|  | CD28− CD8+ T cells | CD3+CD8+CD28- | +1 positive term |  |  |
|  | Effector memory CD4+ T cell | CD3+CD4+CD45RA-CCR7- | +1 positive term |  |  |
|  | CD4+CD28+ T cells | CD3+CD4+CD28+ | -1 negative term |  |  |
|  | CD28+ CD8+ T cells | CD3+CD8+CD28+ | -1 negative term |  |  |
|  | Naive CD4+ T cell | CD3+CD4+CD45RA+CCR7+ | -1 negative term |  |  |
|  | Naive CD8+ T cell | CD3+CD8+CCR7+CD45RA+ | -1 negative term |  |  |

| **Supplementary Table S7.**  **Figure S1 gating-to-phenotype crosswalk** | | | | | |
| --- | --- | --- | --- | --- | --- |
| **Figure panel** | **Gate / reader label** | **Parent denominator** | **Corresponding flow-cytometry phenotype** | **Marker definition** | **Reported denominator** |
| A1 | A1 Lymphocytes | all events | — | — | — |
| A2 | A2 CD3+ T cells | lymphocytes | T cell | CD3+ | % of lymphocyte |
| A3 | A3 CD4/CD8 subsets | CD3+ T cells | CD4+ T cells; CD8+ T cells | CD3+CD4+; CD3+CD8+ | % of T cells; % of T cells |
| B1 | B1 CD4-panel lymphocytes | all events | — | — | — |
| B2 | B2 CD3+ parent gate | lymphocytes | T cell | CD3+ | % of lymphocyte |
| B3 | B3 CD4/CD8 split | CD3+ T cells | CD4+ T cells; CD8+ T cells | CD3+CD4+; CD3+CD8+ | % of T cells; % of T cells |
| C1 | C1 CD8-panel lymphocytes | all events | — | — | — |
| C2 | C2 CD3+ parent gate | lymphocytes | T cell | CD3+ | % of lymphocyte |
| D1 | D1 NK-panel lymphocytes | all events | — | — | — |
| D2 | D2 CD3/CD56 separation | lymphocytes | T-NK cell; NK cell | CD3+CD56+; CD3-CD56+ | % of lymphocyte; % of lymphocyte |
| D3 | D3 NK bright/dim subsets | NK cells | Immature NK cell; Mature NK cell | CD3-CD56+bright; CD3-CD56+dim | % of NK cells; % of NK cells |
| E1 | E1 γδ panel lymphocytes | all events | — | — | — |
| E2 | E2 CD3+ parent gate | lymphocytes | T cell | CD3+ | % of lymphocyte |
| E3 | E3 γδ T cells | CD3+ T cells | γδ T cell | CD3+γδT+ | % of T cells |
| E4 | E4 Vδ1/Vδ2 partition | γδ T cells | Vδ1+ T cell; Vδ2+ T cell; Vδ1+ T cell/Vδ2+ T cell ratio | CD3+γδT+Vδ1+; CD3+γδT+Vδ2+; | % of γδT cells; %of γδT cells; |
| F1 | F1 B-cell panel lymphocytes | all events | — | — | — |
| F2 | F2 CD45+ leukocytes | lymphocytes | — | — | — |
| F4 | F4 CD21/CD38 B-cell gate | B cells | CD21- B cell | CD3-CD19+CD38-CD21- | % of B cells |
|  |  |  |  |  |  |
| Notes: | | | | | |
| 1. The crosswalk is a reader-facing mapping aid for representative gating documentation and does not claim a complete parent-gate hierarchy. | | | | | |
| 2. Only panels submitted as Supplementary Figure S1 are included; support-only panels and G panels were excluded from this submission-facing crosswalk. | | | | | |

| **Supplementary Table S8.**  **Abbreviation list** | | |
| --- | --- | --- |
| **Abbreviation** | **Definition** | **Category / note** |
| PN | pulmonary nodule | core cohort/group abbreviation |
| Adeno-PN | Adeno-spectrum pulmonary nodule, including adenocarcinoma-spectrum PN and small invasive adenocarcinoma presenting through the pulmonary-nodule clinical pathway | core subgroup abbreviation |
| LC | lung cancer | core disease abbreviation |
| Overt LC | pathologically confirmed primary lung cancer managed through the lung-cancer clinical pathway | core group abbreviation |
| NSCLC | non-small cell lung cancer | histology/subset abbreviation |
| HC | healthy controls with no known medical history and normal chest CT findings | control group abbreviation |
| OR | odds ratio | statistical abbreviation |
| CI | confidence interval | statistical abbreviation |
| SD | standard deviation | statistical abbreviation |
| q | FDR-adjusted P value | statistical abbreviation |
| AAH | atypical adenomatous hyperplasia | pathology abbreviation |
| AIS | adenocarcinoma in situ | pathology abbreviation |
| MIA | minimally invasive adenocarcinoma | pathology abbreviation |
| NLR | neutrophil-to-lymphocyte ratio | clinical readout abbreviation |
| MLR | monocyte-to-lymphocyte ratio | clinical readout abbreviation |
| SII | systemic immune-inflammation index | clinical readout abbreviation |
| CEA | carcinoembryonic antigen | clinical readout abbreviation; descriptive only / not robust in this study |
| γδ | gamma-delta | flow-cytometry phenotype notation |
| Vδ1 | V delta 1 | flow-cytometry phenotype notation |
| Vδ2 | V delta 2 | flow-cytometry phenotype notation |
| FDR | false discovery rate | statistical abbreviation |
| IQR | interquartile range | statistical abbreviation |
| BMI | body mass index | clinical covariate abbreviation |
| COPD | chronic obstructive pulmonary disease | clinical covariate abbreviation |
| NK | natural killer | flow-cytometry abbreviation |
| Tfh | T follicular helper | flow-cytometry abbreviation |
| Th | T helper | flow-cytometry abbreviation |
| Tc | T cytotoxic | flow-cytometry abbreviation |
| LUAD | lung adenocarcinoma | histology abbreviation |
| SCLC | small-cell lung cancer | histology abbreviation |
| HLA-DR | human leukocyte antigen DR | marker abbreviation |
| PD-1 | programmed cell death protein 1 | marker abbreviation |
| NKG2D | natural killer group 2D | marker abbreviation |

## Supplementary Figures

## Supplementary Figure S1. Representative flow-cytometry gating strategy. Representative flow-cytometry gating strategy for the submitted Supplementary Figure S1 panels. Gates and percentages were retained from the provided plots; gate boundaries were not redrawn or moved, and percentages/event counts were not edited, recalculated, or retyped. Denominator labels follow the denominator/unit reference table and are used as reader-facing denominator labels. Detailed parent denominators and phenotype definitions are provided in the supplementary tables. FMO controls were used where available; thresholds were otherwise set using negative populations and a laboratory fixed gating template within a unified hierarchical gating strategy. γδ = gamma-delta; Vδ1 = V delta 1; Vδ2 = V delta 2.
